# Love bites: male frogs (*Plectrohyla*, Hylidae) use teeth scratching to deliver sodefrin precursor-like factors to females during amplexus

**DOI:** 10.1186/s12983-021-00445-6

**Published:** 2021-11-25

**Authors:** Lisa M. Schulte, An Martel, Raciel Cruz-Elizalde, Aurelio Ramírez-Bautista, Franky Bossuyt

**Affiliations:** 1 Department of Wildlife-/Zoo-Animal-Biology and Systematics, Faculty of Biological Sciences, Goethe University Frankfurt, Max-von-Laue-Str. 13, 60438 Frankfurt/Main, Germany; 2 Wildlife Health, Ghent University, Salisburylaan 133, 9820 Merelbeke, Belgium; 3 Laboratorio de Zoología, Facultad de Ciencias Naturales, Universidad Autónoma de Querétaro, Avenida de Las Ciencias S/N, Santa Fe Juriquilla, C. P. 76230 Querétaro, Mexico; 4 Laboratorio de Ecología de Poblaciones, Centro de Investigaciones Biológicas, Instituto de Ciencias Básicas E Ingeniería, Universidad Autónoma del Estado de Hidalgo, Km 4.5 carretera Pachuca-Tulancingo, 42184, Mineral de La Reforma, Hidalgo, Mexico; 5 Amphibian Evolution Lab, Biology Department, Vrije Universiteit Brussel (VUB), Pleinlaan 2, 1050 Brussels, Belgium

**Keywords:** Chemical communication, Breeding glands, Amphibia, Amplexus, Allohormone pheromones, Traumatic mating, Sodefrin precursor-like factor

## Abstract

**Background:**

Efficient transfer of chemical signals is important for successful mating in many animal species. Multiple evolutionary lineages of animals evolved direct sex pheromone transmission during traumatic mating—the wounding of the partner with specialized devices—which helps to avoid signal loss to the environment. Although such direct transmission modes of so-called allohormone pheromones are well-documented in invertebrates, they are considered rare in vertebrates. Males of several species of the frog genus *Plectrohyla* (Hylidae, Anura) have elongated teeth and develop swollen lips during the breeding season. Here we investigated the possibility that these structures are used to scratch the females’ skin and apply allohormone pheromones during traumatic mating in several *Plectrohyla* species.

**Results:**

Our behavioural observations revealed that males press their upper jaw onto the females’ dorsum during amplexus, leaving small skin scratches with their teeth. Histological examinations of the males’ lips identified specialized mucus glands, resembling known amphibian pheromone glands. Whole-transcriptome sequencing of these breeding glands showed high expression of sodefrin precursor-like factor (SPF) proteins, which are known to have a pheromone function in multiple amphibian species.

**Conclusions:**

Our study suggests SPF delivery via traumatic mating in several anuran species: the males have specialized breeding glands in the lips for production and secretion and use their elongated teeth as wounding devices for application. We hypothesize that these SPF proteins end up in the females’ circulatory system, where understanding their exact function will require further molecular, physiological and behavioural testing.

**Supplementary Information:**

The online version contains supplementary material available at 10.1186/s12983-021-00445-6.

## Background

The oldest and most widespread intraspecific communication system involves chemical signals and can be found in aquatic as well as terrestrial vertebrates [1, 2]. Chemical signals released in the environment by one individual and detected by specific devices of another, where they induce a specific reaction, are defined as pheromones [3]. However, environmental constraints such as temperature, humidity, wind and water currents influence the longevity and arrival of these signals and therefore can limit the effectiveness of communication via pheromones [2, 4]. Animals developed different solutions to this problem, such as long living carrier proteins which slowly release volatile pheromones (e.g. major urinary proteins in mice [5]), by properly navigating air- and water currents towards a pheromone source (e.g. in moths, crabs, and lobsters; for review see [6]), or by fanning pheromones in the direction of the receiver (e.g. in aquatic newts [7]). Another option is the direct transmission of pheromones, hence avoiding any signal loss to the environment. These kind of pheromones can be transferred directly to the nose of the receiver (e.g. during copulation in anurans [8]) or by wounding the mating partner with specialized devices (i.e. traumatic mating, traumatic secretion transfer [9]). The chemicals transferred during traumatic mating are not classified as regular pheromones, but are defined as primer pheromones [10], allohormones [11, 12] or allohormone pheromones [13]. The latter term, allohormone pheromone, takes into account that these chemicals behave similar to hormones (hence allohormones [11]), but that the same class of molecules may also act through the sensory organs in other species (hence defining them as a subclass of pheromones [2]). Allohormone pheromones are, for example, delivered via love-darts in snails (*Cantareus asperses*), increasing the transmitters paternity [14], or via copulatory setae in earth worms (*Lumbricus terrestris*), increasing the sperm uptake of the receiver [15]. Only a single example is currently known from vertebrates: salamanders of the family Plethodontidae possess mental glands beneath the chin, which have been shown to contain courtship pheromones that positively influence the female´s receptivity [16–18]. While in some species these molecules are transferred through the nose, in other species (e.g. *Desmognathus ochrophaeus*) they are transferred by males scraping them with protruding premaxillary teeth through the female´s skin directly into her circulatory system, i.e. inoculating her with allohormone pheromones [19–21]. The chemical signals used during salamander courtship are typically protein pheromones, used for short-range communication (for an overview of the different types of amphibian pheromones see [22–24]). Although some of these proteins have been shown to have originated early in amphibian evolution and can be found among several urodele and anuran families [8, 25, 26], their transmission via traumatic mating using extra-genital wounding structures [9] is currently only known from these salamanders.

In Neotropical anurans of the genus *Plectrohyla*, breeding males of all species of the *Plectrohyla guatemalensis* group [27, 28] possess swollen upper lips as well as protruding maxillary and premaxillary teeth [29–31]. It has been suggested that this combination may have a similar function as the allohormone pheromone inoculation apparatus of plethodontid salamanders [31]. This hypothesis is supported by few observations of *Plectrohyla* in amplexus, during which the male presses its teeth and swollen upper lips against the top of the head of the female [31, 32], the discovery of a female *P. hartwegi* exhibiting several parallel scratches on her head and back [31], as well as by a study connecting the teeth position with potential pheromone glands in one *Plectrohyla* species [33]. Allohormone pheromones positively influencing the female´s receptivity (e.g. resulting in accelerated oviposition) should be of especially great advantage for mating anurans. Being joined in amplexus for several hours or days [34] makes frogs extremely vulnerable to predation [35, 36] and accelerating this process should largely improve their fitness [37]. Here we investigated the possibility of traumatic mating and potential allohormone pheromone transmission in several *Plectrohyla* species by combining histological, morphological and behavioural data, whole transcriptome sequencing (RNAseq) and molecular phylogenetics (compare Table 1).

**Table 1:** *Plectrohyla* species used for the different methods applied

	*P. sagorum*	*P. matudai*	*P. hartwegi*
Behavioural observations (mating)	✓		
Teeth length comparison between males and females	✓	✓	✓
Distance between male teeth vs. Female scratches	✓		✓
Microscopic structures of lip glands	✓	✓	✓
Lip gland protein expression and phylogenetic analysis	✓	✓	

## Results

### Mating behaviour and extra-genital wounding structures

**Fig. 1:**
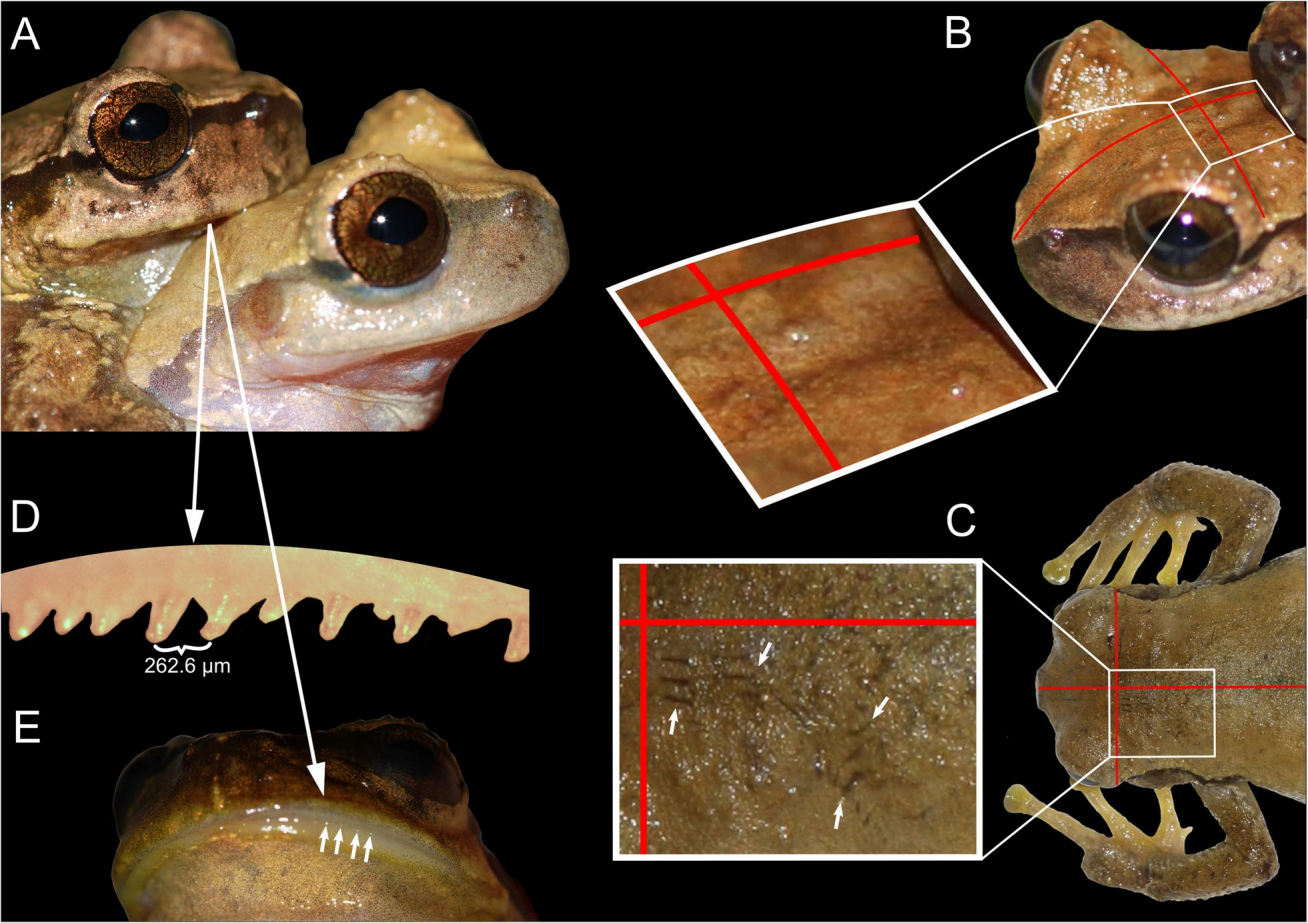
*Plectrohyla sagorum* traumatic mating. **A** Male *P. sagorum* pressing his lips on the female´s head during amplexus. Female skin before (**B**) and after (**C**) the male scratched over her head. White arrows are indicating some of the scratches. **D** Pointed teeth of the male, which are protruding through the swollen lips (**E**; pointed out by white arrows). Comparing the position of the males’ head in (**A**) and (**B**) emphasises the movement of his lips over the females’ dorsum

**Fig. 2:**
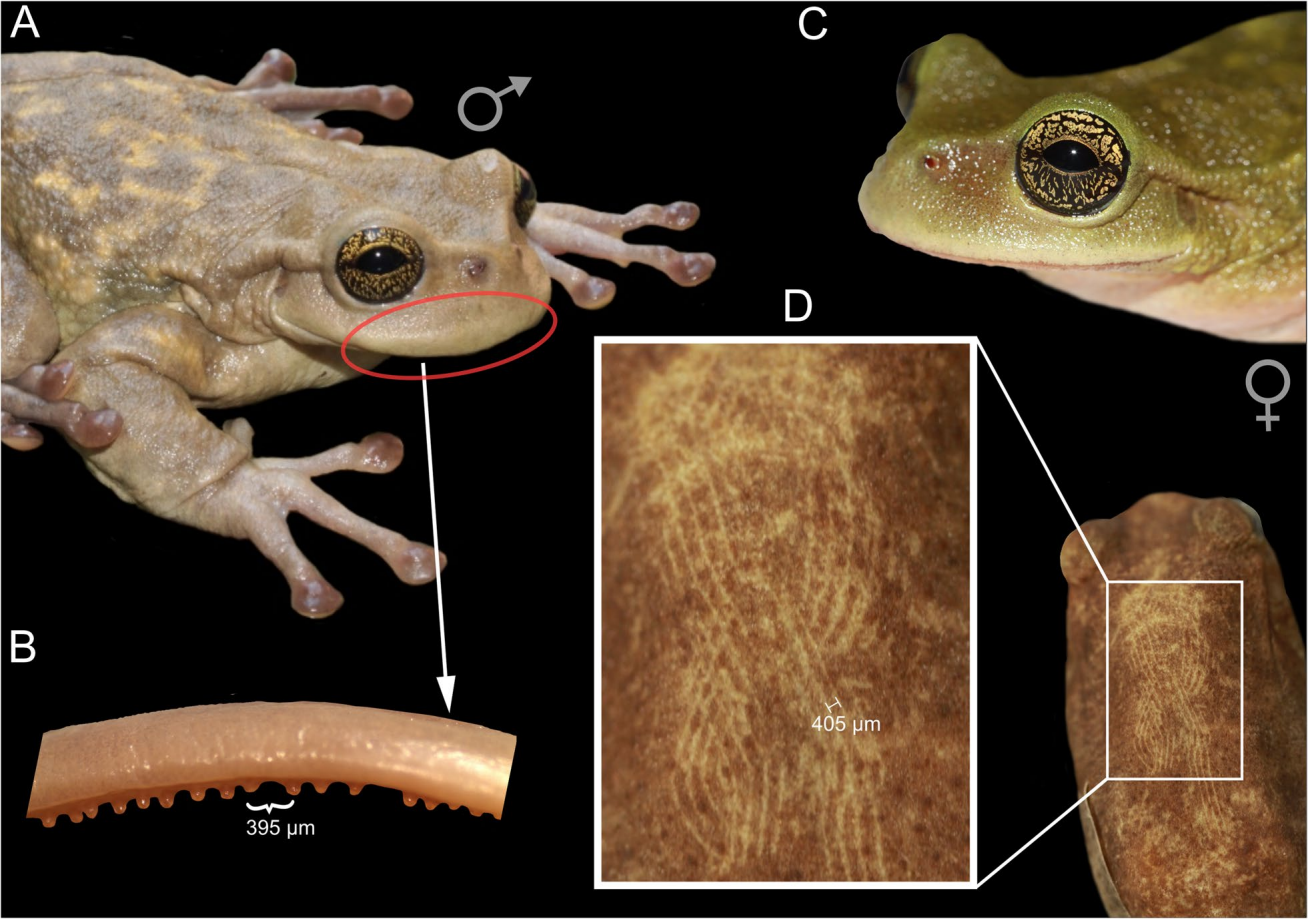
Evidence for traumatic mating in *Plectrohyla hartwegi*. **A** Male *P. hartwegi* with swollen lips. **B** The male’s spatulate teeth protrude through the lips and are most likely used to scratch the female (**C**) during amplexus. **D** shows the potential teeth marks on the back of a female (museum specimen UMMZ 152864)

During field work in Mexico, we kept a couple of *P. sagorum* in amplexus in captivity for 24 h and observed their mating behaviour. During this time, we observed the male pressing its upper jaw against different areas of the female’s dorsum (Fig. 1A, B). After separating and euthanizing both frogs, short parallel scratches were visible on the female´s dorsum (Fig. 1C), which were not present earlier during amplexus (Fig. 1B). When measuring the distances between those scratches parallel to each other (ranging from 184.32 to 402.44 µm; mean = 311.23 ± 69.21 µm) as well as the distances between the male´s upper teeth (ranging from 170.46 to 438.64 µm; mean = 287.48 ± 61.41 µm; Fig. 1D) protruding trough his swollen lips (Fig. 1E), scratches and teeth match up well with each other. The same was the case when we compared the mean distance between the parallel running scars on the dorsum of the female *P. hartwegi* found by Duellman and Campbell [29] (ranging from 332.38 to 558.55 µm; mean = 435.49 ± 69.87 µm) with the teeth distance of a conspecific male (although it was not from the same locality; ranging from 228.56 to 694.76 µm; mean = 387.33 ± 105.41 µm; see also Fig. 2). The well preserved scars of *P. hartwegi*, however, seem to be deeper than those in *P. sagorum*, which after storage in formaldehyde became less visible. This might be correlated either with a difference in intensity of scratching the skin, or with the shape of the teeth in these two species: while *P. hartwegi* has wider spatulate teeth (Fig. 2B), *P. sagorum’s* teeth are pointed (Fig. 1D). In both species, males have on average stronger protruding teeth than conspecific females and the same is the case for another congeneric species *P. matudai* (see Table 2).

**Table 2:** Mean teeth length and mean thickness of the glandular region in the lips of *Plectrohyla* males and females. For males additionally the mean diameter of specialized mucus glands was measured

Species	Sex	Mean teeth length (µm)	Mean thickness of glandular region (µm)	Mean SMG diameter (µm)
*P. hartwegi*	male	189.982 ± 33.819	437.08 ± 27.69	155.77 ± 20.15
	female	129.054 ± 56.743	151.04 ± 13.96	
*P. sagorum*	male	150.868 ± 54.601	144.45 ± 5.45	95.70 ± 4.05
	female	77.818 ± 15.977	71.97 ± 4.42	
*P. matudai*	male	171.469 ± 62.685	142.74 ± 10.19	69.93 ± 17.45
	female	82.391 ± 21.866	89.19 ± 9.18	

### Microscopic structures of lip glands

**Fig. 3:**
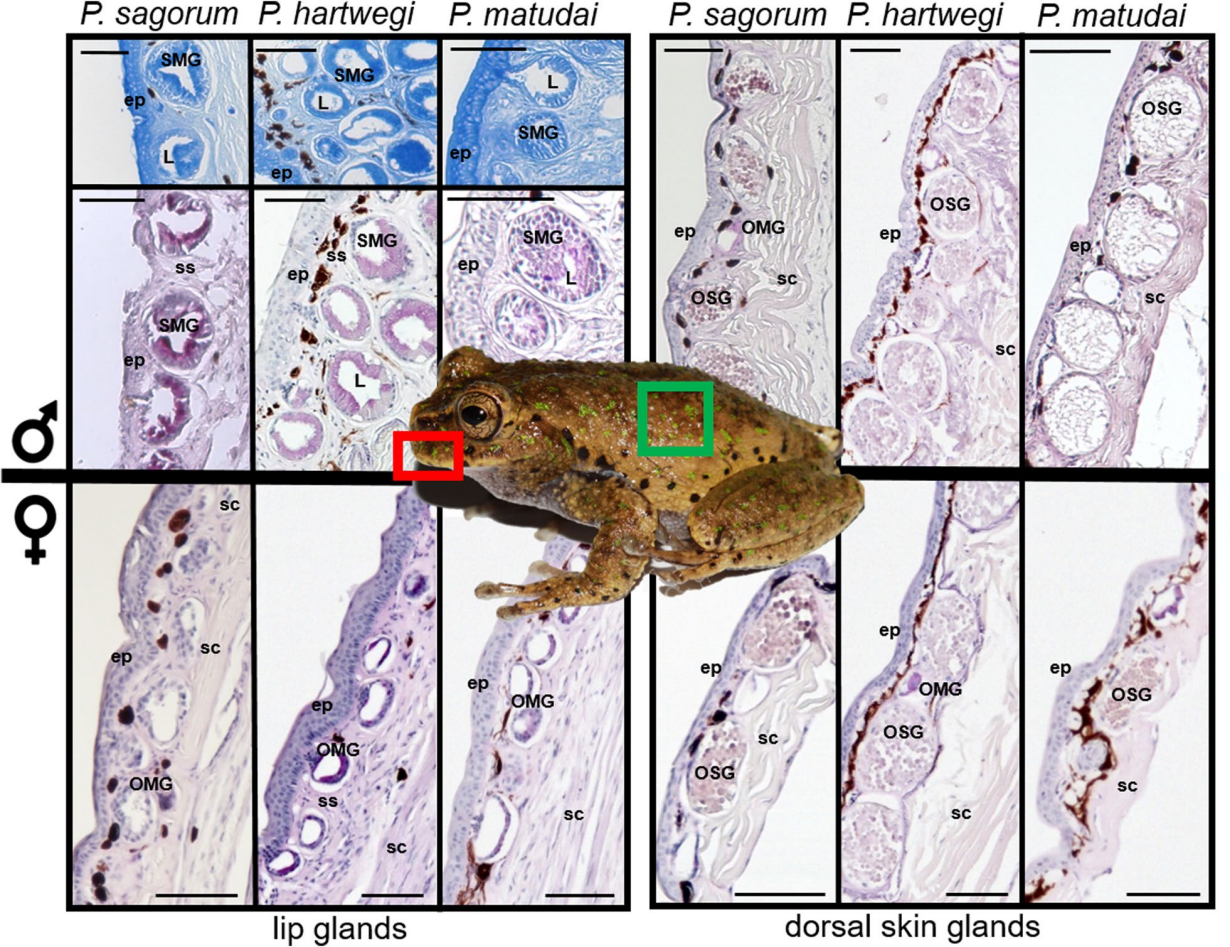
*Plectrohyla* glands. On the left side the upper rows show histological sections through the upper lips (red square) from male *P. sagorum*, *P. hartwegi* and *P. matudai*, the lower row shows the equivalent female sections. The top sections are stained with Coomassie Brilliant Blue R-250, all other sections with periodic acid Schiff. On the right side sections through the dorsal skin (green square) are shown for males (upper row) and females (lower row), all stained with periodic acid Schiff. The frog in the middle of the figure is a male *P. matudai*. ep, epidermis; L, lumen; OMG, ordinary mucus glands; SMG, specialized mucus glands; sc, stratum compactum; ss, stratum spongiosum. Scale bars = 100 μm

Histological examinations of skin samples from the lip and dorsal region of the three examined *Plectrohyla* species show that the dorsal skin in males and females exhibits the usual structure of anuran skin, containing mainly so-called ordinary serous glands, as well as some scattered ordinary mucous glands (see Fig. 3). The glands found in the lips, however, deviate strongly from this pattern. Here no serous glands, but only mucous glands can be found. When comparing the lip sections between sexes, we found that the glandular region is 1.6 to 3 times thicker in males than in females (Table 2). While the female glands are ordinary mucous glands (OMGs), the glands found in the male lips are specialized mucous glands (SMGs), i.e. breeding glands (as defined by [38]). Unlike the rather flattened OMGs, the *Plectrohyla* SMGs are round or longitudinal oval-shaped with a short intra-epidermal duct (where visible). Their secretory portions have radially arranged tall cells (with basal nuclei), projecting into the relatively wide devoid central lumen (Fig. 3). *Plectrohyla hartwegi*, being the biggest of the three examined species, has the largest SMGs, compared to *P. sagorum* and *P. matudai* (Table 2). While the SMGs in this species are organized in several layers, in the other two species they are arranged in a single layer along the epidermis (compare Fig. 3). The glands occur along the inner and outer portion of the lip, with the gland ducts exiting throughout the entire width of the lips with no specific cognizable pattern. Staining reactions to PAS and Coomassie Blue R250 were positive for all SMGs.

### Secretory lip gland protein expression analysis and phylogenetic analysis

**Fig. 4:**
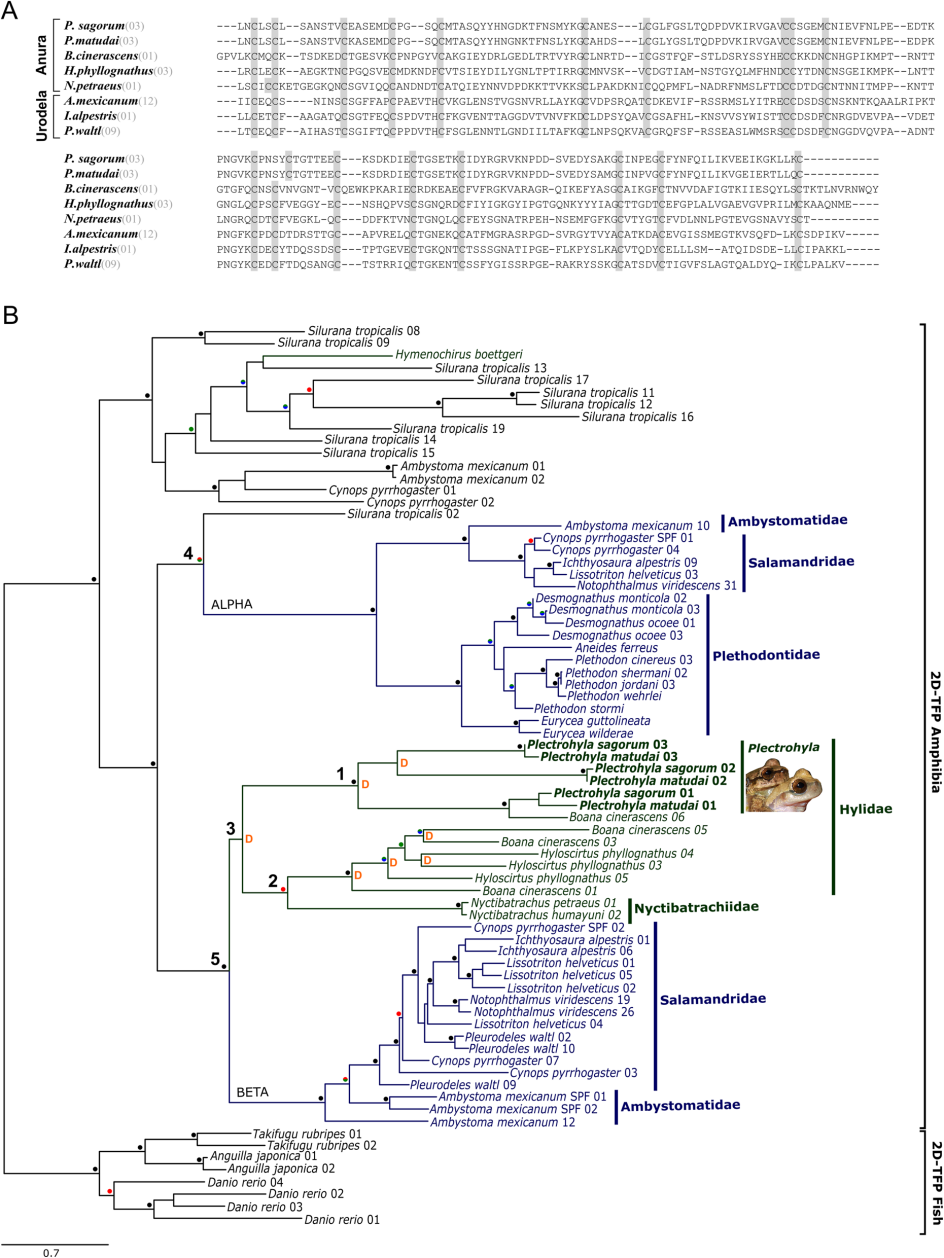
Sequence similarities and evolutionary relationships between SPF proteins. (A) Alignment of selected beta-SPF transcripts (numbers refer to transcripts used in the tree (B)). Protein sequences show a typical 10 + 8-cysteine pattern in the first and second motif, suggesting a high structural similarity (but note the slight deviation of cysteine position 2 in the second motif of the *Plectrohyla* sequences). (B) Maximum likelihood tree of 2-domain-TFPs (2D-TFP) from amphibians and teleost fish (outgroup). Anuran transcripts that originated from breeding glands are shown in green, salamander transcripts from breeding glands in blue (see Additional file 1). Nodes supported by all three assessment methods are indicated with a black circle, nodes only supported by one or two methods are indicated with blue, green and/or red (ultrafast Bootstrap > 95%, red; SH-aLRT > 80%, green; aBayes-support > 0.95, blue). Duplication events within the anuran SPF clade are marked with “D”, other nodes within this clade are considered speciation nodes (Notung analysis). Numbers 1–5 are referred to in the text

To identify the proteins specifically expressed in the gland tissue of the frogs, we conducted whole-transcriptome sequencing (RNA-seq) on the gland-containing swollen upper lips of the males of two of the *Plectrohyla* species. Our results revealed the expression of several proteins that in other animals are known to be involved in egg development, fertilisation, locomotor processes, blood pressure and metabolic regulation (see Table 3). Interestingly, we also found high expression of several two-domain three-finger proteins (2D-TFPs) (*P. sagorum*: 10 different 2D-TFPs, 3 of which make up for 75% of the in total > 17,800 transcripts per million [TPM]; *P. matudai*: 16 different 2D-TFPs, 3 of which make up for 75% of the in total 10,000 TPM) termed sodefrin precursor-like factors (SPFs). These SPFs show protein sequence identities of up to 96% within *Plectrohyla*, while identities with other amphibian SPFs are up to 61% for anurans and up to 41% for salamanders. Structurally, there is a minor variation in the cysteine arrangement of *Plectrohyla* sequences compared to most other amphibian SPF sequences (Fig. 4A), while the rest of the cysteine pattern is identical. Further protein pheromone sequences known from other amphibian species (such as amplexin or plethodontid modulating factor) were not found in the male lips.

**Table 3:** Selection of BLAST homologies found in *Plectrohyla* lip glands and their known functions in other animals. PS = *P. sagorum*, PM = *P. matudai*; **** ≥ 5000, ** ≥ 2500, ** ≥ 1000, * ≥ 500 (in parenthesis = expression level below 50 TPM)

Protein homology	Extract of known functions in other species	Main location of expression in other taxa	Expression level	References
2D-TFPs (e.g. sodefrin precursor-like factor, phospholipase A2 inhibitor)	Sex pheromones, toxins, cholinergic signalling, immune regulation, Phospholipase inhibition	Sex glands; various organs	PS****, PM****	[44, 45, 84]
Kazal-type inhibitors (Ovomucoid-like)	Protease inhibitor (e.g. Trypsin), egg protein, possibly secretion protection	Oviduct (liver)	PS**, PM****	[85–87]
Ovostatin	Protease inhibitor (e.g. Trypsin), egg protein, possibly secretion protection	Oviduct	PS**, PM**	[86–89]
Vitelline membrane outer layer protein I (VMO-I)	Egg membrane protein; ovomucin expression, antimicrobial barrier for eggs	Oviduct	PS**, PM***	[90, 91]
Fish-egg lectin	Egg protein, antimicrobial	Ovary (but also other organs)	PS, PM	[92]
Jeltraxin	Egg protein, binds calcium (pot. Fertilization facilitation)	Oviduct	PS, PM*	[93]
Avidin	Egg protein, binds biotin (pot. Bacterial growth inhibition)	Oviduct	PS, PM	[94]
Cysteine rich secretory proteins (CRISP-familiy; e.g. serotriflin-like protein, MGC108118 protein precursor)	Involved in fertilization (sperm guidance), antimicrobial, venoms	Uterus (some also other organs)	PS***, PM**	[87, 95–98]
Beta-microseminoprotein	Seminal plasma protein, antibacterial, antifungal	Prostate (but also other organs)	PS*, PM*	[99, 100]
Thyrotropin releasing hormone (TRH)	Influence on reproductive behaviour, locomotor activity	Hypothalamus	PM (PS)	[101–103]
Tachykinin	Gut tissue contraction, defensive skin peptide, vasodilation, locomotor stimulation	Various organs	PM (PS)	[104, 105]
Glucagon	Blood sugar elevation, appetite regulation	Pancreas	(PM)	[47, 57, 106, 107]
Nucleobindin 2 peptide (NUCB2)	Fat metabolism regulation, appetite regulation	Spleen, testis and stomach	PS (PM)	[108]
Resistin-like	Peptide hormone derived from adipose tissue, insulin-antagonist	Gastrointestinal tract	PM	[109]
Inactive pancreatic lipase-related protein 1 (PL-RP1)	Inhibitor of dietary triglyceride digestion	Pancreas	PS, PM	[110]

Because SPF proteins are known for their pheromone function in several amphibians [25, 26], we analysed the phylogenetic position of our sequences to get a perspective on whether our SPF sequences fall into the same clades as those with known pheromone function. For this, we aligned our SPF sequences with a broad range of sequences from other amphibians and outgroup teleost fish sequences and performed phylogenetic analyses. Our Maximum Likelihood analyses resulted in a single best tree (-*ln L* = 21,679.364), showing two well-supported clades of salamander pheromones (Fig. 4B, alpha and beta), each grouping with an anuran lineage (both representing speciation events; Fig. 4B, branches 4 and 5, respectively). Our top-expressed sequences of *P. sagorum* and *P. matudai* all fall into a single clade (together with one *Boana* sequence; Fig. 4B, node 1), which is the sister lineage (not strongly supported; presenting a duplication event) of the remaining anuran SPFs in the beta clade (represented by node 2).

## Discussion

It has been suggested that male tree frogs of the *Plectrohyla guatemalensis* species group transfer allohormone pheromones from their lips into the female’s skin by scratching their mating partners with their teeth during amplexus, i.e. via traumatic mating [31, 39]. The results of our study support this hypothesis. In the lips of all three examined species, we identified specialized mucus glands (SMGs), a type of sexually dimorphic skin gland, that has been described in various amphibian species (including another *Plectrohyla* species [33]). In several anurans which bring their SMGs in direct contact with the female´s nares during mating [8] as well as in plethodontid salamanders known for transdermal transmission [40] these glands have been shown to produce proteins that are involved in chemical communication. In *Plectrohyla* the ducts of the SMGs exit through the epidermis onto the inner and outer lips (also see [33]). This way the gland content can be easily applied into the scratches caused by the teeth when the lips are pulled over the females’ skin (also see discussion in [33]). The strong positive staining of the SMGs with Coomassie Brilliant Blue R-250 indicates a proteinaceous content of the glands, and the PAS-positive staining suggests that these proteins may contain carbohydrate chains. Combined, these results hint towards a potential glycoprotein allohormone pheromone content, as previously shown for other breeding glands [8, 41–43].

When analysing the protein content of the glands, we found several proteins whose potential role during reproduction requires further examination (see Table 3), and identified highly expressed sodefrin precursor-like factors in the frog lips as prime allohormone pheromone candidates. The evolution of SPF as a pheromone system has already been well investigated for some amphibians. It is known to have a history of gene duplications of which the derived proteins of two gene lineages (alpha and beta, differing in cysteine patterns, for details see [44]) were shown to perform a pheromone function in salamanders [25, 45]. Furthermore, a recent study showed that multiple families of both terrestrially and aquatically reproducing anurans also independently recruited SPF proteins, most likely for chemical sexual communication. The recruitment of this potential pheromone happened when the evolution of the male breeding gland location and courtship behavior allowed transmission of its secretion to the female’s nares [8]. In our study we compared the SPF proteins found in the *Plectrohyla* lip glands with those nasally delivering anurans as well as the same set of urodelan SPF sequences used before [8], in order to further estimate the evolution of chemical communication roles for SPF. Our phylogenetic tree is congruent with previous analyses of amphibian SPFs [8, 45], showing well-supported clades of alpha and beta salamander pheromones (Fig. 4B, indicated with alpha and beta). Notung analysis confirms that the groupings of each of these salamander clades with an anuran lineage represent the speciation of anurans from salamanders (Fig. 4B, nodes 4 and 5), and therefore represent amphibian alpha and beta clades. While the only anuran SPF sequence not derived from adult gland tissue falls into the alpha clade (*Silurana tropicalis* 02, see Additional file 2), all SPFs extracted from anuran breeding glands (Fig. 4B, marked green) cluster with the beta clade of salamander pheromones. Interestingly, our top-expressed *Plectrohyla* sequences all fall in a clade for which only a single frog SPF was currently known (*Boana cinerascens* 06; Fig. 4B, branch 1). This sequence, according to our Notung analysis, is orthologous with one of each *Plectrohyla* species sequences (*Plectrohyla sagorum* 01 and *Plectrohyla matudai* 01). Also, the other two *Plectrohyla* sequences show orthology between both species (i.e. both sequences 02 and 03, respectively; see Fig. 4B). The clade including all *Plectrohyla* (and one *Boana*) sequences is the sister lineage to all other beta SPFs extracted from anuran breeding glands (Fig. 4B, marked green), i.e. two hylid and two nyctibatrachid species [8]. These analyses thus indicate that our *Plectrohyla* SPF sequences stem from a duplication that happened in anurans (Fig. 4B, node 3) before the divergence of Hyloidea (Nobleobatrachia) from Ranoidea (Natatanura) (Fig. 4B, node 2), i.e. earlier than 155 million years ago [46]. It is possible that this duplication in early anuran evolution represents a co-option of beta-SPF to a new delivery mode or function in chemical communication, but this cannot be confirmed at this point. Our overall results however suggest that, in extant *Plectrohyla,* these SPF proteins are delivered to the female transdermally, thus bypassing the olfactory system.

Our findings entail the intriguing hypothesis that SPF proteins not only recurrently acquired chemical communication functions in amphibians, but that reception by the female also independently evolved both vomeronasal and transdermal routes in salamanders and frogs. Indeed, in salamanders of the family Plethodontidae, SPF is one of three known protein pheromones secreted from specialized breeding glands [47], and transdermal allohormone pheromone delivery through traumatic mating, which can be found in all genera, is the ancestral delivery mode in this family. Delivery by slapping the mental glands onto the nostrils of the female is regarded as a derived mode and has only been found in a single genus (*Plethodon* [48]). Species in this genus are also known to be the only ones secreting the pheromone plethodontid receptivity factor (PRF) in their glands, which appears to be a replacement for SPF in this group [49]. SPF, on the other hand, has been shown to be expressed in the glands of several plethodontid genera, including those exclusively using the ancestral transdermal delivery mode [50]. SPF increases the female receptivity in one of these species (*Desmognathus ocoee*) after diffusion through the skin [51]. In other investigated urodelan families (Salamandridae, Ambystomatidae), transdermal delivery does not exist, and SPF was not replaced, but is delivered through the water or directly onto the nares of the female during amplexus [44, 45]. The same is the case for anuran species known to express SPF in their breeding glands: they either press their glands onto or next to the female nares before and during amplexus, or they wave them through the water towards the female [8]. Although we have some observations regarding the mating behaviour of *Plectrohyla sagorum*, we only saw the male rubbing his teeth and lips over the head and back of the female while they were already in amplexus. We do not know if these frogs additionally present their glands to the female before entering amplexus (e.g. by pressing their inflared lips onto her nares), as has been shown in other hylid species [8, 52]. Due to the strong evidence of scratching behaviours as well as the high SPF expression in the lips which are rubbed through the scratches, we suggest transdermal delivery (i.e. being part of the traumatic mating [9]), which would define SPF as an allohormone pheromone in *Plectrohyla*. A possible effect of SPF in *Plectrohyla* may be the reduction of time spent in amplexus, e.g. by accelerating oviposition. However, an experimental evaluation, e.g. through mating set-ups with and without SPF delivery, is still pending. Besides salamanders and frogs using their teeth for transdermal allohormone pheromone delivery, it has also been suggested that frog species that possess nuptial pads on their fingers (including gland tissue and often spines), use these secondary sexual characters as traumatic mating devices during amplexus [53]. For one species the gland content of the nuptial pads has been described. Even though a potential allohormone pheromone protein found in these glands (called amplexin) shares similarities with a known salamander pheromone, SPF was not found here [54].

In Plethodontidae, it has been observed that the blood vessels of peripheral circulation close to the skin dilate during courtship [55], possibly supporting the transportation of gland secretions [56]. A similar scenario is conceivable for *Plectrohyla* frogs: if the SPF proteins are released directly into the blood stream, they may bypass the need for central processing, and possibly directly bind to tissue- and organ-specific receptors, [13, 57] e.g. at the ovaries. Here they may directly influence physiological processes, such as oviposition. However, the possibility that they are also centrally processed (via different pathways than pheromones delivered over the nares) cannot be ruled out at this point and the identification of (allohormone) pheromone binding sites remains a challenging task for future studies.

### Conclusion

Our study confirms the performance of traumatic mating, i.e. the wounding of females with specialized male devices (here the teeth) in hylids and suggests the delivery of proteins belonging to an early diverged anuran clade of SPFs as allohormone pheromones during amplexus. Although the exact effects of these molecules in anurans remain unknown [8] and although SPF has been shown to evoke various effects in urodelan females (such as following behaviour, cloacal gaping or courtship acceleration [44]), all essentially come down to shortening the courtship duration. Being phylogenetically nested among well-established urodelan SPF pheromones (Fig. 4B), we propose a similar role for the SPFs found in *Plectrohyla*. Yet, their processing after release into the circulatory system and the potential physiological or behavioural changes triggered in the female still await further research.

## Methods

### Study species and collection sites

During August of 2015 we collected adult frogs of three of the 19 species of the *Plectrohyla guatemalensis* group (Hylidae, Hylinae, Hylini) from the Nuclear Central American highlands in Chiapas, Mexico. Twelve male and one female *P. sagorum*, three male and one female *P. matudai* as well as one female *P. hartwegi* were collected in the Sierra Madre de Chiapas. We assured that frogs were in breeding mood, since all males but one *P. sagorum* were calling when we found them. The male that did not call was already in amplexus with a female. The couple was kept in captivity for 24 h and regularly checked before sacrificed. All frogs were euthanized injecting 2% lidocaine (PiSA®). Because *P. hartwegi* males do not have vocal sacs nor vocal slits and therefore do not call [58], we had difficulties to find males of this species. We loaned three males together with the female specimen which was shown to have conspicuous scratches on head and back [31] from the museums. Due to a lack of *P. matudai*-samples for histological sectioning, we further loaned one male specimen of this species, too.

### Teeth morphology and scar analysis

In order to compare between sexes, we measured the length of 17–26 maxillary and premaxillary teeth of each, one male and one female of each of the three species. For this purpose, pictures of the teeth were taken, either by pushing back the upper lip or after removing it for histological analysis (see below). All well visible teeth were measured from the gingiva (not including teeth roots visible through the gum) to the tip of the tooth using the ImageJ1.47t software [59] and the mean teeth length (± SD) was calculated. Using the same measurement methods, we additionally compared the distance between parallel scars found on female’s backs (being suggested to derive from male’s teeth [31]) with the distance between teeth of conspecific males.

### Histological sectioning of lip tissue

To examine the swollen lips histologically, specimens were fixed in 4% formaldehyde and preserved in 70% EtOH after fixation. We removed a ca. 10 mm long and 3 mm wide strip of skin from the upper lip and a ca. 5 × 5 mm sized piece of dorsal skin from the shoulder region as a control. Samples were dehydrated in a graded series of ethanol (70–100%), paraffin-embedded, cut at 5 μm with a rotary microtome and mounted onto microscope slides. All sections were stained with Periodic acid–Schiff (PAS) for the detection of mucins [60], glycogens [61] and neutral glycoproteins [62]. Male lip sections were further stained with Coomassie Brilliant Blue R-250 for detection of proteins [63]. Samples were examined with a Nikon eclipse 90i microscope and images were captured with a Nikon DS-Fi1 camera and processed with NIS-Elements BR3.2. In order to compare the glandular region in the lip between males and females, we measured the thickness from the epidermis to the deepest internal glands on the three widest spots on a random slide, using ImageJ1.47t [59]. We further measured the greatest diameter of the five biggest specialized breeding glands of the males of each of the three species.

### Whole transcriptome sequencing (RNAseq) and expression analysis

To identify potential allohormone pheromone expression, the lips of four freshly euthanized male *P. sagorum* (one of which was in amplexus with a female before, see above) and two *P. matudai* were placed into 1 ml RNAlater (Life Technologies) each and stored at 0–5 °C during transportation and then at -20 °C until RNA isolation. The total RNA (pooled by species) was extracted using the RNeasy Plus Universal Mini Kit (Qiagen). De novo transcriptome sequencing was performed at DNAvision, Gosselies, Belgium. A paired-end cDNA sequencing library (75 bp) was constructed using the Illumina TruSeq RNA sample preparation kit for sequencing on the Illumina HiSeq2000 platform (Illumina, San Diego, California). Adaptor sequences and low quality bases were trimmed using Trim-Galore v0.5.0 [64] allowing a minimum read size of 25 base pairs and a phred score > 33. Low-quality regions were removed with Trimmomatic v0.38 [65], using the default settings and retaining reads with a minimum length of 25 base pairs and a phred score > 20. Rcorrector v1.03 [66] was used for identification and removal of random Illumina sequencing errors. For each sample, we generated three de novo assemblies: two using SPAdes v3.12 with kmer sizes 25 and 53 [67], and one with Trinity v2.8.5 [68]. Subsequently, we merged all three assemblies using the EvidentialGene tr2aacds pipeline [69] in order to reduce the levels of transcript redundancy. To assess the completeness and redundancy of our assemblies, we analysed them before and after merging with BUSCO v3.0.2 [70] (using the vertebrata database) and calculated contigs metrics with TransRate v1.0.3 [71] (see Additional file 2). All transcripts were annotated with the Identical Protein Groups (IPG) database from NCBI, which was filtered to contain only vertebrate sequences, using the Blastx mode of Diamond v0.9.22 [72]. Read alignment and expression calculation was done with Kallisto v0.42.4 [73]. We then manually checked all transcripts for known amphibian protein pheromones (i.e. amplexin, imorin, persuasion, plethodontid modulating factor, plethodontid receptivity factor, silefrin, sodefrin, sodefrin precursor-like factors, splendiferin) and further identified all putative secretory proteins by searching the amino-acid sequences (obtained through transdecoder v5.5.0, imbedded in EvidentialGene) for proteins which either have a predicted signal peptide (identified with SignalP v5.0 under default parameters [74]), or were identified as soluble and extracellular (using DeepLoc-1.0 [75]). All secretory proteins > 50 TPM where then manually categorized, and while those that are typically expressed in (skin) tissue (e.g. house-keeping genes, ribosomal proteins or keratins) were not further pursued, those that stood out for being sex specific (e.g. being usually expressed in sex-specific tissues) or for normally being connected to other organs (e.g. gut tissues), were grouped in functional categories (see Table 3). The total expression values of these groups were calculated by adding up the TPM values.

### Molecular phylogenetics of allohormone pheromone candidates

For those of the *Plectrohyla* species where whole transcriptome sequencing identified highly elevated expression of sodefrin precursor-like factor (SPF) we chose the three highest expressed SPF-amino acid sequences (GenBank accession numbers: OL598420 - OL598425). We combined these sequences with known SPF-protein-sequences from other anurans (Hylidae, Nyctibatrachidae and Pipidae) and urodeles (Salamandridae, Plethodontidae, Ambystomatidae) as well as a set of genomic sequences of 2-domain-TFPs of amphibians and teleost fish (see Additional file 1; also compare data used in [8]). The group of fish sequences were chosen as outgroup [45, 76]. After comparison of different alignment programs and settings, sequence alignment was done in MAFFT v.7 using the L-INS-I method [77]. Phylogenetic relationships were estimated under maximum likelihood with IQ-TREE [78] using FLU + F + R4 as best-fitted model assigned by ModelFinder [79] To assess branch support at each node we used the ultrafast bootstrap function with 1000 replicates (UFBoot2; [80]), as well as SH-like approximate likelihood ratio tests (SH-aLRT, 1000 replicates; [81]) and approximate Bayes tests (aBayes; [82]). A speciation-duplication analysis was performed using Notung 2.9 [83].

## Supplementary Information

**Additional file 1**. Species, accession numbers and tissue-origin of the 2D-TFP sequences used in the maximum likelihood tree (Fig. 4).

**Additional file 2**. Statistics of the de novo transcriptome assemblies performed with the lip tissue RNA samples from *P. matudai* and *P. sagorum*.

## Data Availability

The raw reads generated and analysed during the current study have been deposited in the European Nucleotide Archive (ENA) under project accession number PRJEB4879.
