# Supplementary material for: Love bites: male frogs (Plectrohyla, Hylidae) use teeth scratching to deliver sodefrin precursor-like factors to females during amplexus
Source: Front Zool. 2021 Nov 25;18:59. doi: 10.1186/s12983-021-00445-6 (PMC8613984; doi:10.1186/s12983-021-00445-6)
Supplement: Supplementary file 1 — Additional file 1. Species, accession numbers and tissue-origin of the 2D-TFP sequences used in the maximum likelihood tree (Fig. 4). [file 12983_2021_445_MOESM1_ESM.docx]

**Supplementary material**

| **Additional file 1.** Accession numbers and tissue-origin of sequences in phylogeny | | |
| --- | --- | --- |
| Species | Accession number | Tissue |
| Ambystoma mexicanum 01 | CN040015 | larval limb tissue |
| Ambystoma mexicanum 02 | CN044610 | larval limb tissue |
| Ambystoma mexicanum 10 | KU043462 | courtship-water (cloaca-gland) |
| Ambystoma mexicanum 12 | KU043460 | courtship-water (cloaca-gland) |
| Ambystoma mexicanum SPF 01 | KU043451 | courtship-water (cloaca-gland) |
| Ambystoma mexicanum SPF 02 | KU043455 | courtship-water (cloaca-gland) |
| Aneides ferreus | AAZ06335 | mental-gland |
| Anguilla japonica 01 | GAGT01010462 | gill |
| Anguilla japonica 02 | GAGT01006841 | gill |
| Boana cinerascens 01 | MK457706 | mental gland |
| Boana cinerascens 03 | MK457708 | mental gland |
| Boana cinerascens 05 | MK457710 | mental gland |
| Boana cinerascens 06 | MK457711 | mental gland |
| Cynops pyrrhogaster 01 | FS299680 | lens regenerating iris |
| Cynops pyrrhogaster 02 | FS301877 | ovary |
| Cynops pyrrhogaster 03 | KU213617 | abdominal-gland |
| Cynops pyrrhogaster 04 | KU213618 | abdominal-gland |
| Cynops pyrrhogaster 07 | KU213621 | abdominal-gland |
| Cynops pyrrhogaster SPF 01 | KU213615 | abdominal-gland |
| Cynops pyrrhogaster SPF 02 | KU213616 | abdominal-gland |
| Danio rerio 01 | XM_003200278 | n.a. |
| Danio rerio 02 | XM_003200388 | n.a. |
| Danio rerio 03 | XM_002665247 | n.a. |
| Danio rerio 04 | XP_002665292 | n.a. |
| Desmognathus monticola 02 | AAZ06326 | mental-gland |
| Desmognathus monticola 03 | AAZ06327 | mental-gland |
| Desmognathus ocoee 01 | AAZ06329 | mental-gland |
| Desmognathus ocoee 03 | AAZ06332 | mental-gland |
| Eurycea guttolineata | AAZ06338 | mental-gland |
| Eurycea wilderae | AAZ06337 | mental-gland |
| Hyloscirtus phyllognathus 03 | MK457714 | mental gland |
| Hyloscirtus phyllognathus 04 | MK457715 | mental gland |
| Hyloscirtus phyllognathus 05 | MK457717 | mental gland |
| Hymenochirus boettgeri | MK457705 | postaxillary gland |
| Ichthyosaura alpestris 01 | KP849562 | abdominal-gland |
| Ichthyosaura alpestris 06 | KP849567 | abdominal-gland |
| Ichthyosaura alpestris 09 | KP849570 | abdominal-gland |
| Lissotriton helveticus 01 | KJ402326 | abdominal-gland |
| Lissotriton helveticus 02 | KJ402327 | abdominal-gland |
| Lissotriton helveticus 03 | KJ402328 | abdominal-gland |
| Lissotriton helveticus 04 | KJ402329 | abdominal-gland |
| Lissotriton helveticus 05 | KJ402330 | abdominal-gland |
| Notophthalmus viridescens 19 | AIT39225 | cloaca-gland |
| Notophthalmus viridescens 26 | AIT39232 | cloaca-gland |
| Notophthalmus viridescens 31 | KP118902 | cheek-gland |
| Nyctibatrachus humayuni 02 | MK457719 | finger-gland |
| Nyctibatrachus petraeus 01 | MK457718 | finger-gland |
| Plethodon cinereus 03 | AAZ06284 | mental-gland |
| Plethodon jordani 03 | AAZ06317 | mental-gland |
| Plethodon shermani | AAZ06311 | mental-gland |
| Plethodon stormi | DQ097067 | mental-gland |
| Plethodon wehrlei | ABD34631 | mental-gland |
| Pleurodeles waltl 02 | KM463923 | cloaca-gland |
| Pleurodeles waltl 09 | KM463930 | cloaca-gland |
| Pleurodeles waltl 10 | KM463931 | cloaca-gland |
| Silurana tropicalis 02 | CR761728 | gastrula embryos |
| Silurana tropicalis 08 | B1H2M6 | brain |
| Silurana tropicalis 09 | XP_002942613 | liver and blood |
| Silurana tropicalis 11 | F7DSQ4 | n.a. (expressed in different organs) |
| Silurana tropicalis 12 | F6SPH0 | n.a. (expressed in different organs) |
| Silurana tropicalis 13 | F6PQG9 | n.a. (expressed in different organs) |
| Silurana tropicalis 14 | F6Q7B9 | n.a. (expressed in different organs) |
| Silurana tropicalis 15 | XP_002942715 | n.a. |
| Silurana tropicalis 16 | F7DT47 | n.a. (expressed in different organs) |
| Silurana tropicalis 17 | F6YM66 | n.a. (expressed in different organs) |
| Silurana tropicalis 19 | F7C6U1 | n.a. (expressed in different organs) |
| Takifugu rubripes 01 | XP_003966124 | n.a. |
| Takifugu rubripes 02 | XP_003966053 | n.a. |
