# Supplementary material for: Love bites: male frogs (Plectrohyla, Hylidae) use teeth scratching to deliver sodefrin precursor-like factors to females during amplexus
Source: Front Zool. 2021 Nov 25;18:59. doi: 10.1186/s12983-021-00445-6 (PMC8613984; doi:10.1186/s12983-021-00445-6)
Supplement: Supplementary file 2 — Additional file 2. Statistics of the de novo transcriptome assemblies performed with the lip tissue RNA samples from P. matudai and P. sagorum. [file 12983_2021_445_MOESM2_ESM.docx]

| **Additional file 2**. De novo transcriptome assembly statistics | | | | | | | | | | | | | | | | | | |  | |  | |  | |  | |  | |
| --- | --- | --- | --- | --- | --- | --- | --- | --- | --- | --- | --- | --- | --- | --- | --- | --- | --- | --- | --- | --- | --- | --- | --- | --- | --- | --- | --- | --- |
|  |  | | |  |  | |  | | |  | |  |  | | |  | | |  | |  | |  | |  | |  | |
|  | |  | | | BUSCO* | | | | | | | |  | |  | | Transrate contig metrics | | | | | | | | | | |  |
|  | | |  | | | complete  (%) | | duplicated (%) | fragmented (%) | | missing  (%) | | |  | number of contigs (n) | | | mean lenght | | with ORF | | mean ORF% | | > 1000 bases | | n50 | | optimal score |
| *P.matudai* | | | SPAdes 25k | | | 2058 (79.6) | | 295 (11.4) | 303 (11.7) | | 225 (8.7) | | |  | 157856 | | | 530,95 | | 21127 | | 52.09 | | 24677 | | 1806 | | 0.1113 |
|  | | | SPAdes 53k | | | 1428 (72.4) | | 446 (17.2) | 367 (14.2) | | 345 (13.4) | | |  | 132430 | | | 590,09 | | 20935 | | 55.96 | | 21447 | | 1621 | | 0.0761 |
|  | | | Trinity | | | 2129 (82.4) | | 700 (27.1) | 212 (8.2) | | 245 (9.4) | | |  | 1191138 | | | 867,04 | | 27614 | | 56.32 | | 29499 | | 1630 | | 0.0903 |
|  | | | Evigene | | | 2196 (84.9) | | 38 (1.5) | 147 (5.7) | | 243 (9.4) | | |  | 57320 | | | 913,50 | | 15334 | | 58.42 | | 16402 | | 1737 | | 0.2711 |
| *P. sagorum* | | | SPAdes 25k | | | 2051 (79.3) | | 284 (11.0) | 313 (12.1) | | 222 (8.6) | | |  | 168266 | | | 529,08 | | 21673 | | 51.11 | | 26115 | | 1821 | | 0.0025 |
|  | | | SPAdes 53k | | | 1921 (74.3) | | 545 (21.1) | 342 (13.2) | | 323 (12.5) | | |  | 222574 | | | 431,61 | | 24321 | | 52.80 | | 27386 | | 2205 | | 0.0026 |
|  | | | Trinity | | | 2145 (83.0) | | 721 (27.9) | 196 (7.6) | | 245 (9.4) | | |  | 126679 | | | 880,53 | | 28660 | | 55.11 | | 31937 | | 1665 | | 0.0004 |
|  | | | Evigene | | | 2120 (82.0) | | 43 (1.7) | 196 (7.6) | | 270 (10.4) | | |  | 45335 | | | 1132,80 | | 14948 | | 55.78 | | 17102 | | 1980 | | 0.2246 |
| *Total BUSCO groups searched: 2586 | | | | | | | | | | | | | | | |  | | |  | |  | |  | |  | |  | |
